# Aortic Non-coronary Cusp Perforation Following Methicillin-Sensitive Staphylococcus aureus (MSSA) Endocarditis

**DOI:** 10.7759/cureus.84897

**Published:** 2025-05-27

**Authors:** Rea Isaac, Dhara Rana, Aobo Li, Vakula Atthota, Benjamin Silverman

**Affiliations:** 1 School of Medicine, Rowan-Virtua School of Osteopathic Medicine, Stratford, USA; 2 Department of Internal Medicine, Inspira Health Network, Vineland, USA; 3 Department of Infectious Disease, Inspira Health Network, Vineland, USA; 4 Department of Cardiology, University of Pennsylvania, Vineland, USA

**Keywords:** aortic endocarditis, aortic pseudoaneurysm, aortic valve, mssa endocarditis, non-coronary cusp of aortic valve, pseudoaneurysm

## Abstract

Isolated perforation of the aortic valve’s noncoronary cusp (NCC) is an exceptionally rare but serious complication following bacterial endocarditis. Here we present a unique case of a 62-year-old male who developed an isolated NCC perforation after recovering from Methicillin-sensitive *Staphylococcus aureus *(MSSA) endocarditis. Despite successful antibiotic therapy, the patient experienced progressive cardiac symptoms, ultimately leading to the discovery of severe aortic insufficiency due to NCC perforation. This case underscores the importance of vigilant follow-up and the potential for late-onset complications in patients with a history of endocarditis.

## Introduction

Isolated perforation of the aortic valve’s noncoronary cusp (NCC) is an exceptionally rare but serious complication following bacterial endocarditis. While endocarditis often leads to valve damage and subsequent complications such as regurgitation and perforation, the occurrence of an isolated NCC perforation post-treatment is scarcely documented. Infective endocarditis is a relatively uncommon condition with an incidence of three to 10 cases per 100,000 people in the United States of America [1]. Common endocarditis-associated pathogens include *Staphylococcus aureus*, gram-positive streptococci, staphylococci, and enterococci infections. [1]. Of these pathogens, *S. aureus *remains the main pathogen, accounting for around 30% of endocarditis cases [1]. Infectious endocarditis has many sequelae of complications, one of which includes valve vegetations leading to downstream insufficiency, regurgitation, and even perforation [1]. Of these complications, isolated perforation of the aortic valve NCC following a course of treated bacterial endocarditis is rare. Given the rarity of isolated NCC perforation, most information is derived from individual case reports, and comprehensive epidemiological data are lacking [2,3]. Here, we describe a rare case of a patient who presented with isolated perforation of the noncoronary cusp of the aortic valve following resolved Methicillin-sensitive *Staphylococcus aureus *(MSSA) endocarditis.

## Case presentation

A 62-year-old African American male with a past medical history of congestive heart failure, hypertension, non-insulin-dependent diabetes mellitus, obstructive sleep apnea, morbid obesity, psoriasis, and poor dental hygiene was hospitalized for around a month for severe septic shock secondary to MSSA bacteremia. Additional social history includes that he was a retired engineer and married, but did not visit the dentist frequently. During the first admission in April, there was suspicion of endocarditis. Due to the suspicion for endocarditis during this admission, a transthoracic echocardiography (TTE) showed possible aortic valve vegetation noted at the base of the non-coronary cusp and a 1 to 2 cm moderate pericardial effusion. After the initiation of antibiotics to treat the MSSA bacteremia, repeat blood cultures were negative. The patient was treated with IV cefazolin for 6 weeks, outpatient. 

One month later, in May (second admission), the patient presented to the hospital with dyspnea and chest pain. Cefazolin was continued during this hospital stay. The patient had a stress test, which showed apical thinning but no ischemia with a normal ejection fraction (EF). To further investigate the apical thinning on the stress test, Cardiology recommended a left heart catheterization to rule out ischemic cardiomyopathy. The patient had a left heart catheterization, which showed 80% proximal stenosis of the right coronary artery. Due to his history of endocarditis, blood cultures were drawn and were negative. He was discharged and placed on metoprolol succinate 25 mg, isosorbide mononitrate 30 mg, and furosemide 40 mg daily.

Another month later, in June (third admission), the patient was admitted to the hospital for another episode of chest pain. The patient underwent left heart catheterization, which revealed 99% stenosis of the ostial right coronary artery and was stented.

The patient presented to the outpatient cardiology office for follow-up with symptoms of increased shortness of breath with exertion, leading to the fourth admission in September. On the physical exam, the patient had a significant diastolic heart murmur. The TTE showed an EF of 55% to 60%, normal left ventricular wall thickness, and a grade II LV diastolic dysfunction. The aortic valve revealed an eccentric jet of aortic insufficiency against the anterior mitral valve leaflet, suggesting severe aortic insufficiency. The tricuspid valve showed mild to moderate regurgitation. Due to these new findings on the follow-up appointment, the patient was told to obtain a TEE to investigate the aortic valvular vegetation within a month. This follow-up TEE showed a normal left ventricular EF of 60% to 65% and no thrombus in the left atrial appendage. The aortic valve demonstrated perforation of the non-coronary cusp, causing severe aortic regurgitation. The mitral valve revealed moderate mitral valve regurgitation, and the tricuspid valve showed moderate tricuspid regurgitation. Only trace pulmonic regurgitation was seen, but there was no evidence of a pericardial effusion, as well as an average aortic root size (Video 1). 

**Video 1:** Transthoracic echocardiography (TTE) showing perforation of the non-coronary cusp causing severe, anteriorly directed aortic regurgitation

**Figure 1:**
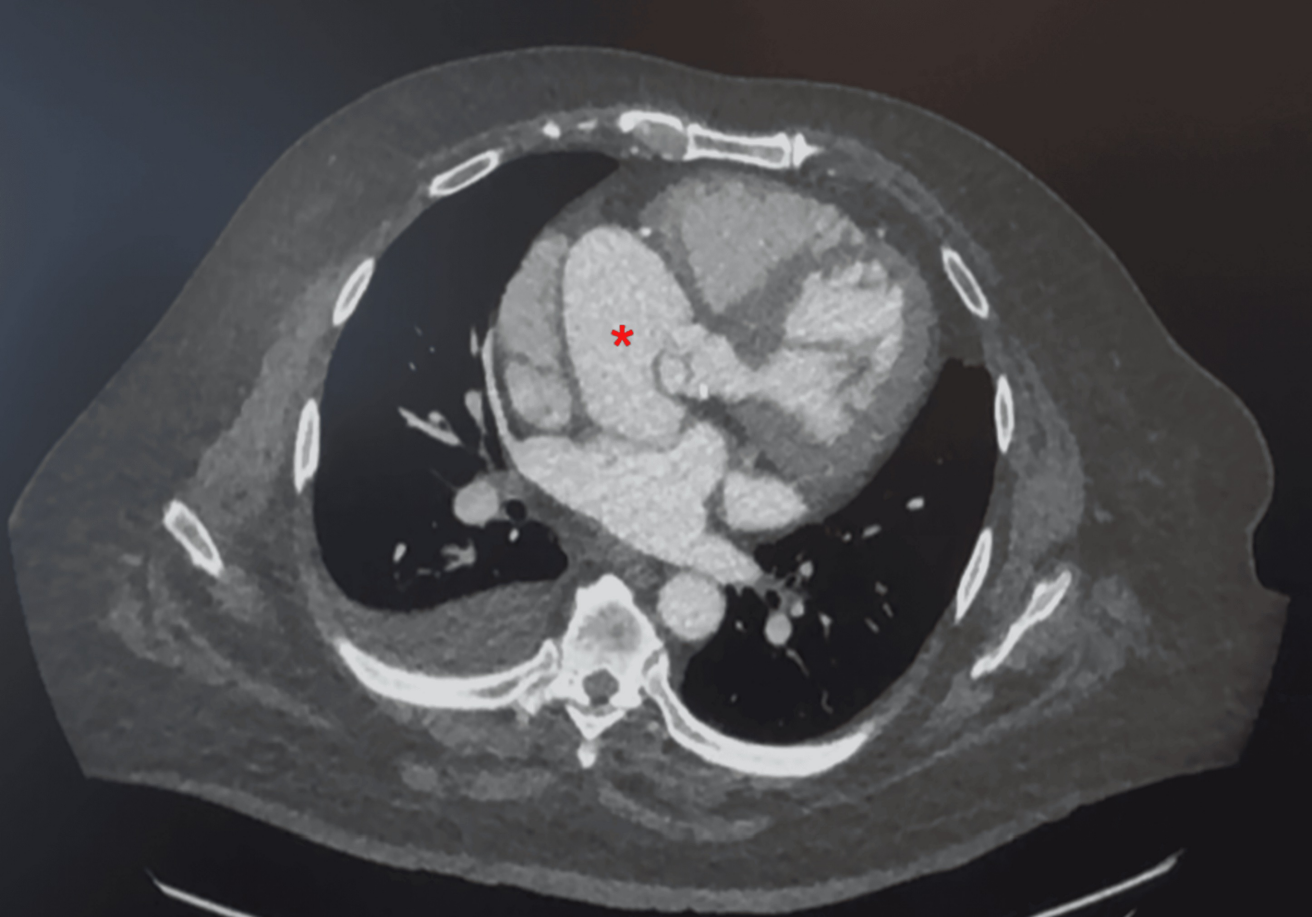
CT angiography thorax showed a large aortic root abscess/pseudoaneurysm, measuring 6.2 cm × 4.4 cm × 5.6 cm and the neck of the pseudoaneurysm measured 17 × 13 mm (indicated by the red *).

After the TEE findings, the patient was referred to CT surgery for evaluation. During the CT evaluation visit, the patient presented with chest pain, dyspnea on exertion, and orthopnea. The patient stated that his symptoms worsened over the last couple of months. He could not perform his activities of daily living. On the physical exam, the patient had lower extremity edema. The thorax CT angiography, soon after visiting the surgeon, showed a large aortic root abscess/pseudoaneurysm arising from the left ventricular outflow tract (LVOT) just inferior to the junction of the right and noncoronary sinus of Valsalva (Figure 1). The pseudoaneurysm measured 6.2 cm × 4.4 cm × 5.6 cm, and the neck of the pseudoaneurysm measured 17 × 13 mm. There was mild calcification of the left aortic valve cusp and moderate calcification of the aortic annulus. 

Given the CT findings, the patient was directly admitted to a community hospital for a non-coronary cusp perforation causing severe aortic insufficiency. The patient was then transferred to a tertiary care center for subsequent surgical management. Pre-intervention TEE showed a large pseudoaneurysm at the aortic root arising from a communication at the level of the LVOT at the right coronary cusp (RCC)/non-coronary cusp (NCC) commissure.

He underwent an aortic root valve replacement, annular reconstruction with a patch, aortic root replacement with homograft, and a coronary artery bypass graft × 1. Post-intervention TEE revealed a homograft aortic valve (AV) leaflet appearing normal with no regurgitation or stenosis, EF remained unchanged at 60%, and a pseudoaneurysm was no longer seen.

The polymerase chain reaction (PCR) tissue culture of the aortic valve was negative for gram stain, anaerobic, fungal, acid-fast bacteria, and broad-range bacterial polymerase chain reaction. The blood cultures three days after the operation were negative. The patient's postoperative course was complicated by a complete heart block and atrial fibrillation with rapid ventricular rate. The patient self-cardioverted to sinus rhythm, and amiodarone 200 mg was started. The patient was then discharged with a peripherally inserted central catheter (PICC) line for six weeks of ceftriaxone.

The patient was seen at a 2-week outpatient cardiology follow-up, and a TTE was performed. It showed normal left ventricle (LV) size with mildly reduced systolic function, left ventricular ejection fraction (LVEF) 45% to 50%, and the right ventricle appeared mildly dilated with mildly reduced systolic function. The bioprosthetic aortic valve was well-positioned with a transaortic valve gradient measuring 10 mmHg, aortic valve area (AVA) 2.2 cm^2^, which is normal in the setting of bioprosthetic valves. He had no bioprosthetic aortic valve regurgitation or pericardial effusion. The mitral valve revealed trace mitral regurgitation. The patient remained stable and symptom-free. The patient continued his 6-week course of IV ceftriaxone via a PICC line.

## Discussion

One of the extremely rare complications of endocarditis is isolated perforation of the non-coronary cusp of the aortic valve. Interestingly, our patient presented with a perforated NCC and had an aortic pseudoaneurysm starting from the LVOT to the RCC/NCC. In a review of the literature by Kunal et al, researchers found 25 cases of aortic cuspal aneurysms. Only four of the 12 cases comprised NCC aneurysms, which was similar to our patient's presentation [2]. None of the cases in the study presented with a perforation of the NCC. Our patient’s course was unique in that he had both an NCC perforation and an RCC/NCC aneurysm. 

The cause of cusp perforation can be due to iatrogenic causes, consequences of resolved endocarditis, or the result of a resection of a papillary fibroelastoma [2]. A proposed mechanism is that infective endocarditis of the AV worsens the preexisting valvular tissue injury, causing the weakening of the aortic cusp, and leading to a subsequent perforation [2]. Potential ways to reduce patient suffering from this condition include prompt diagnosis and symptom management. Our patient initially had aortic valve vegetation three months prior to the presentation of the cusp perforation. He was treated with a 6-week antibiotic course to treat the initial MSSA bacteremia. We propose that after the resolved MSSA endocarditis, the aortic valve cusp tissue was left significantly weakened. Therefore, this increases the chances of cusp perforation from the shear force that the aortic valve withstands.

The classic presentation of cusp perforation is aortic insufficiency [4]. This complication was seen in our patient’s course. TEE is the best diagnostic tool to determine the mechanism of aortic insufficiency [4]. Proper treatment of cusp perforation includes patch repair of the wall defect [4]. Our patient’s perforation was managed with an aortic root valve replacement, annular reconstruction with a patch, and an aortic root replacement with homograft.

## Conclusions

Although infectious endocarditis has many sequelae, one of the complications of endocarditis is an isolated perforation of the noncoronary cusp of the aortic valve. Our case report highlights the significance of the potential for a perforation following healed endocarditis. Early diagnosis and treatment, such as in our case, is vital in preventing patient mortality and the possibility of severe cardiac events. 

## References

[1] Yallowitz AW, Decker LC (2025 Jan-). Infectious endocarditis. StatPearls [Internet].

[2] Kunal S, Shah B, Bagarhatta R, Verma H (2022). Perforated cuspal aneurysm of aortic valve following infective endocarditis presenting as complete heart block: a case report and review of literature. Eur Heart J Case Rep.

[3] Fowler NO, Hamburger MH, Bove KE (1967). Aortic valve perforation. Am J Med.

[4] David TE (2013). Cusp repair in aortic valve procedures: advanced techniques. Tex Heart Inst J.

